# Astrocyte–Neuron Networks: A Multilane Highway of Signaling for Homeostatic Brain Function

**DOI:** 10.3389/fnsyn.2018.00045

**Published:** 2018-11-27

**Authors:** Sara Mederos, Candela González-Arias, Gertrudis Perea

**Affiliations:** Department of Functional and Systems Neurobiology, Instituto Cajal (IC), CSIC, Madrid, Spain

**Keywords:** astrocytes, behavior, neuron–glia signaling, synaptic plasticity, heterogeneity

## Abstract

Research on glial cells over the past 30 years has confirmed the critical role of astrocytes in pathophysiological brain states. However, most of our knowledge about astrocyte physiology and of the interactions between astrocytes and neurons is based on the premises that astrocytes constitute a homogeneous cell type, without considering the particular properties of the circuits or brain nuclei in which the astrocytes are located. Therefore, we argue that more-sophisticated experiments are required to elucidate the specific features of astrocytes in different brain regions, and even within different layers of a particular circuit. Thus, in addition to considering the diverse mechanisms used by astrocytes to communicate with neurons and synaptic partners, it is necessary to take into account the cellular heterogeneity that likely contributes to the outcomes of astrocyte–neuron signaling. In this review article, we briefly summarize the current data regarding the anatomical, molecular and functional properties of astrocyte–neuron communication, as well as the heterogeneity within this communication.

## Introduction

A fundamental property of the mammalian brain is its ability to modify its function based on experience, and thereby to alter subsequent behavior. By changing the strength of transmission at preexisting synapses, transient experiences can be incorporated into the neuronal circuits as persistent memory traces during both development and adulthood. As such, synaptic plasticity is a fundamental mechanism that supports brain function (Buzsáki and Chrobak, 2005). Among the different factors that regulate synaptic plasticity, glial cells have been found to be key players in maintenance of synapse homeostasis (Eroglu and Barres, 2010). The biggest challenge when studying the effects of glial cells on brain activity is isolating the different cell-type components, i.e., neurons vs. glia. Recent research advances using various strategies, such as pharmacological or genetic manipulation and gene expression from viral vectors (Nimmerjahn and Bergles, 2015; Oliveira et al., 2015; Ben Haim and Rowitch, 2017), have allowed researchers to elucidate the role of glial cells in several aspects of brain function, and such knowledge may lead to the development of new therapies and biomarkers for many types of neurological dysfunction (Almad and Maragakis, 2018).

Astrocytes, after oligodendrocytes, constitute the major glial cell population in the mammalian brain (Herculano-Houzel et al., 2015). Since the tripartite synapse concept emerged in 1999 (Araque et al., 1999), data from numerous studies have supported the notion that astrocytes are involved in tight regulation of synaptic transmission (Eroglu and Barres, 2010; Perea et al., 2014; Bazargani and Attwell, 2016). Given that astrocytes have been revealed as strategic cells for controlling neuronal activity, it is crucial to understand the properties and functions of these cells. Astrocytes are now recognized as a markedly heterogeneous group comprising different morphologically specialized cells, such as protoplasmic astrocytes, fibrous astrocytes, perivascular glia, velate astrocytes, Müller cells or Bergman glia, which show particular molecular profiles and which have been extensively reviewed previously (Matyash and Kettenmann, 2010; Reichenbach et al., 2010; Farmer and Murai, 2017). Additionally, there are significant differences between human astrocytes and their rodent counterparts, i.e., the gene expression pattern (Zhang et al., 2016), size and complexity of cellular architecture (Oberheim et al., 2009), and faster calcium dynamics (Oberheim et al., 2009) indicate specialization of glial cells in the human brain that may contribute to the distinctive neurological capabilities that make humans different from other mammals (Han et al., 2013). It is not yet quite clear how those differences account for the higher functions of the human brain (Min et al., 2012; Vasile et al., 2017).

One of the key factors that regulates intracellular signaling in astrocytes is calcium (Ca^2+^). However, because the controversies regarding astrocyte Ca^2+^ signaling and synaptic plasticity, which have been revised in recent excellent reports (Araque et al., 2014; Rusakov, 2015; Fiacco and McCarthy, 2018; Savtchouk and Volterra, 2018), Ca^2+^ signals will not be further discussed in this review article.

The goal of the present review is to highlight the existing data supporting the critical roles of astrocytes in synaptic function, and how those roles may be determined by structurally and functionally different astrocyte populations.

## Ion Homeostasis and Neurotransmitter Uptake

Astrocytes tightly enwrap neuronal cell bodies, axons, dendrites and synapses (Montagnese et al., 1988; Ventura and Harris, 1999; Khan et al., 2001; Witcher et al., 2010), and their endfoot processes associate with vascular endothelial cells and pericytes (Liebner et al., 2018), being ideally positioned to monitor and regulate both synaptic activity and blood brain barrier. The close association between astrocytes and neuronal synapses is a critical factor required for the maintenance of brain homeostasis (Perea et al., 2009). Astrocytes predominantly show potassium (K^+^) conductance (Kuffler and Nicholls, 1966; Hertz et al., 2013), which is mainly due to the inwardly rectifying K ^+^ (Kir) channels that control the hyperpolarized resting potential of astrocytes (Seifert et al., 2009). Among other important channels/ion transporters (e.g., aquaporin-4, chloride channels, Na^+^-Ca^2+^ exchangers; Haj-Yasein et al., 2011b; Halnes et al., 2013), high densities of Kir4.1 channels have been found on thin processes that face the synapses, thus allowing rapid uptake of K^+^ from the synaptic cleft and redistribution of K^+^ in the extracellular space during neuronal activity (Kuffler and Nicholls, 1966; Seifert et al., 2018). Indeed, reduced levels of Kir4.1 protein expression in astrocytes lead to elevated extracellular levels of K^+^ and neuronal membrane depolarization, which has been related to multiple sclerosis, amyotrophic lateral sclerosis, epilepsy and Huntington’s disease (Haj-Yasein et al., 2011a; Jiang et al., 2016; Dossi et al., 2018). K^+^ buffering has been well studied in the retina, where Müller cells show an enriched distribution of Kir channels in endfoot processes (Newman, 1984, 1993). The water channel aquaporin-4 is also highly expressed at the same subcellular domains (Nagelhus et al., 1999), indicating that K^+^ uptake generates parallel water fluxes that are required to dissipate such osmotic changes. Additionally, it has been shown in optic nerve and hippocampus that Na^+^/K^+^-ATPase activity efficiently contributes to the clearance of K^+^ following neuronal activity (Ransom et al., 2000; D’Ambrosio et al., 2002; Larsen et al., 2014), indicating that astrocytes may use a combination of different mechanisms to control extracellular K^+^ levels.

Astrocytic membranes are enriched in glutamate and gamma-aminobutyric acid (GABA) transporters that are differentially expressed throughout the adult brain. These transporters serve as an efficient mechanism for clearing these neurotransmitters (NTs) from the extracellular space after neuronal activity (Borden, 1996; Bergles and Jahr, 1997; Danbolt, 2001). In fact, the expression of glutamate transporter 1 (GLT-1) and glutamate–aspartate transporter (GLAST) prevents glutamate-derived excitotoxicity during neuronal regular synaptic transmission (Danbolt, 2001); and under glutamatergic over-excitation, such as that observed in conditions like epilepsy or brain trauma (Tanaka et al., 1997; Goodrich et al., 2013). Although these transporters are distributed throughout the brain, the highest levels of GLT-1 are found in the hippocampus and the neocortex; while GLAST is enhanced in the cerebellum (Chaudhry et al., 1995; Lehre and Danbolt, 1998), and retina (Rauen et al., 1996; Lehre et al., 1997). Additionally, two populations of astrocytes have been described, based on the predominant expression of particular glutamate transporters in the hippocampus (Matthias et al., 2003). Interestingly, by modulating the expression levels and surface diffusion of glutamate transporters, astrocytes influence synaptic transmission by controlling the glutamate spillover beyond the synapse. Such glutamate spillover can activate extrasynaptic metabotropic glutamate receptors (Huang et al., 2004), which shape the kinetics of excitatory postsynaptic currents (EPSCs; Murphy-Royal et al., 2015). Hence, changes in EPSCs have important effects on the local and temporal integration of synaptic inputs by neuronal networks, and consequently on synaptic plasticity. Therefore, glutamate transporters not only support synaptic homeostasis, but also contribute, at least in part, to plasticity processes at the synaptic levels (reviewed by Rose et al., 2017).

Interestingly, the GABA transporters (GATs) GAT-1 and GAT-3 show particular cellular and sub-cellular distributions throughout the brain (Ribak et al., 1996; Boisvert et al., 2018). GAT-3 is the most abundant GAT in astrocytes and is localized in astrocytic processes that are adjacent to synapses and cell bodies, but are also close to basal and apical dendrites (Boddum et al., 2016), while GAT-1 can be found in distal astrocytic processes and is more abundant in neurons (Borden, 1996; Scimemi, 2014). Activation of GAT-3 results in a rise in Na^+^ concentrations in hippocampal astrocytes and a consequent increase in intracellular Ca^2+^ through the action of Na^+^/Ca^2+^ exchangers (Doengi et al., 2009). Thus, GABA-uptake by astrocytic GAT-3 can stimulate the release of ATP/adenosine that contributes to downregulation of the excitatory synaptic transmission, and provides a mechanism for homeostatic regulation of synaptic activity in the hippocampus (Boddum et al., 2016). In the thalamus, GAT-1 and GAT-3 occupy different domains within the astrocytic membrane, with GAT-1 being located closer to synaptic contacts than GAT-3 (Beenhakker and Huguenard, 2010); this implies that these transporters might play different roles in GABAergic synaptic function. For instance, research suggests that GAT-1 reduces GABA spillover from the synaptic cleft, while GAT-3 controls the extrasynaptic GABA tone, thus regulating tonic inhibition (Beenhakker and Huguenard, 2010). There is a causal relationship between intracellular Ca^2+^ levels and GAT-3 expression in striatal astrocytes (Yu et al., 2018). Downregulation of Ca^2+^ signaling enhances membrane expression of GAT-3, resulting in the reduction of GABAergic tone and abnormal repetitive behavioral phenotypes in mice (Yu et al., 2018) that are related to human psychiatric disorders.

Together with glutamate and GABA uptake, a transient increase in intracellular Na^+^ concentration occurs (Gadea and López-Colomé, 2001a,b). That Na^+^ local boost can be buffered through gap junctions to neighboring astrocytes acting as an intercellular signaling molecule (Rose and Ransom, 1997; Kirischuk et al., 2007). Considering that Na^+^ is also co-transported with other transmitters and molecules, changes in the intracellular Na^+^ concentration are directly related with changes in synaptic transmission (Karus et al., 2015), and the activity of Na^+^/K^+^-ATPase, linking Na^+^ homeostasis to metabolic functions in astrocytes (for review see Chatton et al., 2016).

Therefore, astrocytes are powerful regulators of synaptic activity by combining the extent of synapse coverage and the expression level of ion channels and neurotransmitter transporters at their cell membrane.

Nevertheless, it is important to note that astrocytes do not ensheath all synapses (Ventura and Harris, 1999; Witcher et al., 2010; Chung et al., 2015a). Moreover, the astrocytic coverage of synapses is a highly dynamic process that changes throughout development and adulthood (Chung et al., 2015a; Heller and Rusakov, 2015). Thus, in layer IV of the somatosensory cortex in adult mice, 90% of excitatory synapses are in contact with astrocytes (Bernardinelli et al., 2014a), as compared to 60%–90% of these synapses in the hippocampus (Ventura and Harris, 1999). In the cerebellum, only *ca*. 15% of mossy fiber synapses on granule cells are in contact with astrocyte processes; in contrast, the climbing fibers show *ca*. 85% of synapses covered by astroglial processes, and *ca*. 65% of parallel fiber synapses are also in relatively close contact with Bergmann glia (Xu-Friedman et al., 2001). Additionally, changes in astrocyte–synapse associations can be induced by different neuronal activity levels (Genoud et al., 2006; Bernardinelli et al., 2014b; Perez-Alvarez et al., 2014) and by a range of physiological conditions, including starvation and satiety (Panatier et al., 2006; Theodosis et al., 2008; Chung et al., 2015a). Hence, structural changes in the astrocytic processes can greatly impact the glial network signaling as well as its relationship with synapses, which will shift the function of neuronal circuits.

## Astrocyte Networks

Astrocytes are enriched in gap junctions, which are formed by connexins (Cxs; Nagy et al., 1999). Cx43 and Cx30 are the main Cxs expressed by astrocytes (Nagy et al., 1999). Through gap junctions, which allow intercellular diffusion of ions, second messengers and small molecules of up to *ca*. 1.8 kDa (Kumar and Gilula, 1996), astrocytes form broad cellular networks that involve hundreds of astrocytes (Giaume et al., 2010; Pannasch et al., 2011). In fact, astrocytic intercellular diffusion has been reported for cyclic AMP, inositol-1,4,5-trisphosphate (InsP3), Ca^2+^, glutamate, ATP and energy metabolites (glucose, glucose-6-phosphate and lactate; Tabernero et al., 2006; Harris, 2007). Prior research has demonstrated that the intact function of local astrocyte networks is critical for complex cerebral functions, including sleep–wake cycle regulation, sensory functions, cognition and behavior (for a review see Oliveira et al., 2015; Charvériat et al., 2017). Interestingly, such astrocytic networks show selective and preferential coupling, meaning that not all neighboring astrocytes are functionally connected by gap junctions (Houades et al., 2008; Roux et al., 2011). Based on data of intracellular loading of tracers/reporters in single cells, it has been shown that astrocytes occupy non-overlapping territories, that is, they have independent domains that are established during development (Bushong et al., 2002; Ogata and Kosaka, 2002). However, it remains unclear whether the preferential connectivity between subsets of astrocytes is determined by a common astrocyte progeny during embryonic development or by local factors. Studies focused on astrocyte lineage have revealed that multiple astrocyte clones derived from single precursor cells coexist in the adult cortex, where these clones establish spatially restricted domains that contain up to 40 astrocytes (García-Marqués and López-Mascaraque, 2013). Cx43 is expressed from early in development in radial glial cells; however, Cx30 is expressed postnatally in rodent astrocytes around the third postnatal week (Kunzelmann et al., 1999; Nagy and Rash, 2000). Such different expression of Cxs generates additional differences in the intercellular connectivity of astrocyte networks, with implications in metabolic states (glucose and lactate supply) and synaptic transmission (Rouach et al., 2008). Moreover, gap-junction connectivity is highly sensitive to changes in phosphorylation/dephosphorylation pathways, intracellular calcium levels, pH and redox-related variations (Sáez et al., 2014). Altogether, the data support the existence of plasticity within astrocyte networks. Because astrocytes form large circuits, further studies are required to understand how signals detected within particular astrocytic domains work either locally to affect a few synapses from the same neuron, or remotely to regulate synapses that possibly belong to different neurons or circuits. Future research should also clarify the molecular mechanisms underlying the complex actions of astrocyte–synapse communication in brain circuits.

## Astrocytes: Master Regulators of Synaptic Activity

Intracellular Ca^2+^ signals, driven by endogenous signaling or neuronal activity, are also related to the release of active substances, called gliotransmitters (GTs), which target the synapse via vesicular-dependent (Araque et al., 2000, 2014; Bezzi et al., 2004; Bowser and Khakh, 2007; Parpura and Zorec, 2010) and -independent mechanisms (Duan et al., 2003; Hamilton and Attwell, 2010; Lee et al., 2010; Woo et al., 2012). Although there are controversies regarding the astrocytic expression of different components required for vesicular transmitter release (Schwarz et al., 2017; Bohmbach et al., 2018), several studies have elucidated the mechanisms underlying the dynamic regulation of synaptic transmission by astrocyte activity; this topic has been extensively reviewed (Araque et al., 2014; Bazargani and Attwell, 2016; Allen and Eroglu, 2017).

By releasing glutamate, D-serine, GABA, ATP, adenosine, or tumor necrosis factor-alpha, among others, astrocytes control the basal tone of synaptic activity and the threshold for synaptic plasticity (Beattie et al., 2002; Angulo et al., 2004; Fellin et al., 2004; Jourdain et al., 2007; Perea and Araque, 2007; Henneberger et al., 2010; Bonansco et al., 2011; Di Castro et al., 2011; Panatier et al., 2011; Chen et al., 2013; Shigetomi et al., 2013; Gómez-Gonzalo et al., 2015; De Pittà and Brunel, 2016; Petrelli et al., 2018). One hippocampal astrocyte ensheaths approximately 120,000 synapses (Bushong et al., 2002) belonging to different cell types (excitatory vs. inhibitory neurons) and circuits, and that astrocyte might be able to detect the NTs released from all of those synapses. Indeed, glutamatergic synaptic activation of astrocytes stimulates the release of glutamate, D-serine, ATP, or adenosine, which, through the activation of pre- and postsynaptic receptors sets the threshold for basal synaptic transmission (Bonansco et al., 2011; Panatier et al., 2011), and enhances short- and long-term glutamatergic synaptic plasticity (Jourdain et al., 2007; Perea and Araque, 2007; Henneberger et al., 2010). GABAergic activity stimulates astrocyte Ca^2+^ signaling (Mariotti et al., 2016, 2018; Perea et al., 2016), which induces the release of ATP and adenosine, decreasing the excitatory synaptic tone (Serrano et al., 2006; Covelo and Araque, 2018). Interestingly, hippocampal astrocytes can contribute to neuronal information processing by decoding GABAergic synaptic activity based on frequency and duration of interneuron firing (Perea et al., 2016; Covelo and Araque, 2018). Such decoding dictates whether astrocytes release either glutamate, which enhances excitatory synaptic activity (Perea et al., 2016), or ATP/adenosine, which reduces excitatory synaptic strength (Covelo and Araque, 2018).

Pyramidal cell activity can also engage astrocytes through endocannabinoid (eCB) signaling. eCBs play a critical role in short- and long-term plasticity at both excitatory and inhibitory synapses, mainly via retrograde signaling (Kano, 2014). However, growing evidence indicates that astrocytes participate in eCB signaling, with the postsynaptic activity-dependent release of eCBs stimulating Ca^2+^ signaling in surrounding astrocytes, ultimately influencing glutamatergic synaptic transmission (Navarrete and Araque, 2010; Min and Nevian, 2012; Gómez-Gonzalo et al., 2015; Martín et al., 2015; Andrade-Talavera et al., 2016; Martin-Fernandez et al., 2017; Robin et al., 2018). In fact, research has shown that eCB-astrocyte activation stimulates the release of glutamate, which enhances synaptic strength, with both short-term (Navarrete and Araque, 2010; Martín et al., 2015; Martin-Fernandez et al., 2017) and long-term effects (Gómez-Gonzalo et al., 2015). Moreover, D-serine is released in response to eCB-astrocyte activation, and by stimulating synaptic N-methyl-D-aspartate receptors (NMDARs), actively contributes to hippocampal long-term potentiation (LTP; Robin et al., 2018) and spike timing-dependent long-term depression (tLTD; Min and Nevian, 2012; Andrade-Talavera et al., 2016). Therefore, eCBs that mainly depress synaptic transmission can, by activating astrocytes, exert opposite or additive effects on excitatory synaptic transmission in different brain areas, such as the hippocampus. This has important homeostatic effects that contribute to achieving coordinated activity among neuronal ensembles. Another important factor released by astrocytes is S100β, a Ca^2+^ binding protein, that is able to induce neuronal bursting and engages rhythmic activity both in the dorsal part of the trigeminal main sensory nucleus (NVsnpr; Morquette et al., 2015), and in the prefrontal cortex (Brockett et al., 2018). Additionally, astrocytic S100β enhances synchrony between theta and gamma cortical oscillations and improves cognitive flexibility (Brockett et al., 2018), indicating the behavioral impact of GTs.

These representative examples show the complex and refined effects of astrocyte-released transmitters on neuronal activity. Nevertheless, it is important to keep in mind that astrocytes can also respond to other neuromodulators, such as norepinephrine (Bekar et al., 2008; Paukert et al., 2014), acetylcholine (Takata et al., 2011; Chen et al., 2012; Navarrete et al., 2012; Papouin et al., 2017), dopamine (Jennings et al., 2017), and molecules derived from the neuroendocrine system (Fuente-Martin et al., 2013; Kim et al., 2014), possibly in different fashions depending on the nature of NTs and affecting particular neural circuits that rule behavioral outputs. For example, astrocytes in the hypothalamus respond to the hormones leptin, ghrelin, and insulin, and regulate neuronal activity by releasing ATP (Kim et al., 2014; García-Cáceres et al., 2016), controlling the food consumption. Astrocytes form the dorsal suprachiasmatic nucleus (SCN) and show an anti-phase oscillatory activity compared to neurons, being more active during the night and reducing neuronal firing by the release of glutamate (Brancaccio et al., 2017). Hence, SCN astrocytes show high Ca^2+^ activity at night and release high levels of glutamate into the extracellular space, activating presynaptic NMDARs in SCN neurons, which in turn increases the GABAergic tone across the circuit. However, during daytime extracellular levels of glutamate are reduced by an increased glutamate uptake, and consequently GABAergic tone is reduced, facilitating neuronal firing (Brancaccio et al., 2017). Astrocytes also participate in sleep homeostasis, which is regulated by the accumulation of adenosine (Halassa et al., 2009; Brown et al., 2012). By releasing ATP/adenosine and glutamate, astrocytes regulate cortical states and induce the transition into slow neuronal oscillations associated with sleep (Fellin et al., 2009; Poskanzer and Yuste, 2016; Clasadonte et al., 2017). In this spirit, the lymphatic-like pathway organized by astrocytes and blood vessels in the central nervous system, the “glymphatic” hypothesis (Xie et al., 2013; Plog and Nedergaard, 2018), suggests a significant impact of astrocyte activity during sleep in terms of the clearance of different solutes accumulated during wakefulness. Additionally, glymphatic system seems to be critical for the distribution of nutrients and metabolic homeostasis throughout the brain (Lundgaard et al., 2017), and an enhanced glymphatic clearance has been related with the reduced lactate levels in the brain that usually accompany the transition from wakefulness to sleep (Lundgaard et al., 2017). Therefore, the opposite and complementary neuron–astrocyte signals mutually support the mammalian circadian clock.

Interestingly, the disruption of the glymphatic system has been related with the accumulation of toxic species in the brain, such as amyloid β (Xie et al., 2013). Glymphatic system dysfunctions have been found in murine models that resemble human type 2 diabetes, which also show accumulation of misaggregated proteins (Jiang et al., 2017). Whether glymphatic system alterations and the accumulation of waste in the paravascular space drive the cognitive deficits associated with Alzheimer Disease (AD) or diabetes (Yaffe et al., 2004; Moheet et al., 2015) is under debate (Bacyinski et al., 2017).

## Plasticity at Astrocyte–Neuron Networks

Along with the changes noted in synapses, astrocytes are also sensitive to plasticity processes. Indeed, structural changes, based on the number of synapses covered by astrocyte processes, have been reported in the hippocampus, hypothalamus and cerebellum (Haber et al., 2006; Lippman et al., 2008; Theodosis et al., 2008). Structural imaging studies have shown that fine astrocyte processes have a high motility rate, changing their shape at a time-scale of minutes (Haber et al., 2006; Bernardinelli et al., 2014b; Perez-Alvarez et al., 2014), and can be influenced by learning paradigms, i.e., LTP protocols (Bernardinelli et al., 2014b; Perez-Alvarez et al., 2014). Moreover, after sustained afferent inputs, astrocytes display functional changes based on up/down regulation of membrane ion channels, and neurotransmitter receptors and transporters, showing similar plasticity phenomena to their neuronal counterparts. After using protocols that induce neuronal LTP, hippocampal astrocytes (Pita-Almenar et al., 2006, 2012) show enhanced ability to take up glutamate from adjacent synapses. *In vivo*, whisker stimulation that stimulates LTP in somatosensory cortical neurons also induces an increase of the expression of GLAST and GLT1 in cortical astrocytes (Genoud et al., 2006). In contrast, sustained depression of glutamate transporter currents and AMPA-mediated currents are expressed by Bergmann glia at low frequencies, which typically trigger LTP in Purkinje neurons (Bellamy and Ogden, 2006; Balakrishnan and Bellamy, 2009; Wang et al., 2014). Functional changes are seen not only in terms of neuron-to-astrocyte signaling, that is, the capability of astrocytes to sense and respond to neuronal activity, but also in terms of astrocyte-to-neuron communication. Thus, astrocytes from the ventrobasal (VB) thalamus are capable of adapting their actions on thalamic neurons when protocols for synaptic plasticity are applied to both the peripheral somatosensory and corticothalamic glutamatergic inputs (Pirttimaki et al., 2011). Repetitive stimulation of those pathways leads to a sustained increase in glutamate release from astrocytes, which persists for several minutes after the offset of the stimulus (*ca*. 60 min). Such enhanced gliotransmission affects the nearby thalamic neurons through NMDA receptor activation for long periods, boosting the time window for synaptic plasticity (Pirttimaki et al., 2011). These facts indicate that astrocytes are endowed with mechanisms that allow them to integrate synaptic information and store it for a period of time; therefore, astrocytes are able to memorize synaptic events that will have an impact on subsequent neuronal activity. Hence, astrocytic plasticity is an activity-dependent and input-specific process that is tightly controlled by synaptic activity. However, concomitantly neuronal signaling is dynamically modulated by the surrounding astrocytes, reinforcing the concept that brain function relies on interdependent neuron–astrocyte signaling.

## Astrocyte Heterogeneity

To improve our understanding of brain circuits, it is essential to identify the properties and functions of each of their components. Neurons consist of several subtypes that are defined by their morphology, genetic profile, electrophysiological properties and input/target regions (Bota and Swanson, 2007). Recent data indicate that astrocytes are also a highly heterogeneous cell group with precise neural circuit specializations, especially when considering the wide range of transporters, membrane receptors, protein expression and functions that they exhibit (Zhang and Barres, 2010; Freeman and Rowitch, 2013; Khakh and Sofroniew, 2015). For example, a recent study on astrocyte diversity, which employed state-of-the-art optical, anatomical, electrophysiological, transcriptomic and proteomic approaches, revealed that dorsal striatal and hippocampal astrocytes (stratum radiatum) show significant differences in the sizes of their barium-sensitive K^+^ currents, as well as differences in the spontaneously and synaptically evoked G protein-coupled receptor-mediated Ca^2+^ signals (Chai et al., 2017). Interestingly, hippocampal and striatal astrocytes show different territory sizes, with the territory size being larger for striatal astrocytes, although hippocampal astrocytes display significantly greater and closer physical interactions with excitatory synapses than do astrocytes in the striatum (Chai et al., 2017). Striatal astrocytes are enriched for expression of the aldehyde dehydrogenase 5 family member A1 (Aldh5a1), a protein involved in GABA degradation, which seems highly relevant to a circuit mainly composed of GABAergic medium spiny neurons (Chai et al., 2017). Likewise, astrocytes from the dorsal striatum show functional selectivity in terms of neuronal cell-type activity by responding with variations in Ca^2+^ to the signaling of a particular type of medium spiny neuron (D1 or D2; Martín et al., 2015). By releasing glutamate, astrocytes activated by D1 or D2 neurons will specifically signal only the same type of neuron, implying that astrocyte–synapse signaling is largely cell-type specific (Martín et al., 2015). Compared with the striatum, hippocampal astrocytes are enriched for expression of glial fibrillary acidic protein (GFAP), Cx 43 and glutamine synthetase (Chai et al., 2017), which are likely involved in both glutamate metabolism and astrocyte connectivity in a circuit with strong oscillatory activity. Astrocytes from another dopaminergic nucleus, the ventral tegmental area (VTA), also show specific features that differentiate them from cortical and hippocampal astrocytes. VTA astrocytes show morphological differences, smaller somata and less tissue coverage by their processes, as well as electrical membrane property differences, and reduced expression of Kir4.1 channels (Xin et al., 2018). Furthermore, although gap junction coupling between astrocytes and oligodendrocytes is also present in the hippocampus and cortex, it is significant higher in the VTA region (Xin et al., 2018), which could impact the metabolic states of the dopaminergic neurons and their axons that exhibit tonic firing activity.

Remarkably, one of the molecular markers usually used to identify astrocytes, GFAP, shows different isoforms (α, β, γ, δ and κ) that are variably expressed in astrocytes across different brain regions (Middeldorp and Hol, 2011). Indeed, the cortex shows limited detectable levels of GFAP-labeled astrocytes, mostly located in layer 1 and in deep layers; as well as in the thalamus and other subcortical regions. In contrast, the hippocampus displays a high number of astrocytes expressing detectable levels of GFAP, which is considered to indicate astrocytic molecular diversity. Additionally, developmental and regional differences can be found in terms of the expression of the GLTs GLT-1 and GLAST, which show a dominant expression in different astrocyte populations (Regan et al., 2007). Hence, much effort has been expended in quantitative analysis of the molecular profiles of astrocytes in different brain regions. An integrated transcriptional analysis has been performed, taking advantage of some of the most common proteins expressed by astrocytes, such as GFAP, aquaporin-4, S100β, glutamine synthetase, GLT-1 and Aldh1L1 (Bachoo et al., 2004; Zhang et al., 2016; John Lin et al., 2017; Morel et al., 2017). In this spirit, the astroglial mRNA expression patterns have been examined along the dorsoventral axis, including the cortex, hippocampus, thalamus, hypothalamus, caudate-putamen and nucleus accumbens. These studies revealed opposite profiles between dorsal (cortex and hippocampus) and ventral (thalamus and hypothalamus) regions, i.e., the extracellular matrix protein, secreted protein acidic and rich in cysteine (SPARC) is selectively highly expressed in the hypothalamus/thalamus, while its levels are very low in the cortex/hippocampus (Morel et al., 2017). Additionally, astrocytes promote neurite growth and synaptic maturation of neurons from the same region, that is, subcortical neurons develop larger neurites when they are co-cultured with astrocytes from subcortical regions than with cortical astrocytes (Morel et al., 2017), which suggest that astrocyte modulation of synaptogenesis and synaptic activity is determined by neuronal cell type (Christopherson et al., 2005), but also specific brain areas (Morel et al., 2017).

It is important to establish whether astrocytes located at specific layers within a cortical circuit express different properties. Neurons display layer-specific subtypes that play particular roles in cortical circuitry. Therefore, it is possible that astrocytes show similar layer segregation to support and regulate such circuitry. A recent study on the somatosensory cortex found that, compared to astrocytes in deeper layers, astrocytes located in the upper layers differentially express several molecules related to morphogenesis, synaptic regulation and metabolism (Lanjakornsiripan et al., 2018). Astrocytes from layer 2/3 occupy a larger volume than do astrocytes at layers 4–6 and 1, likely due to greater astrocytic process arborization, thus supporting the notion that astrocytes in different layers possess distinct morphological features. Similarly, astrocytes located at layer 2/3 show more-extensive ensheathment of the synaptic clefts than do astrocytes in layer 6 (Lanjakornsiripan et al., 2018). Additionally, functional differences between astrocytes from different cortical layers have been described *in vivo* (Takata and Hirase, 2008). Astrocytes located in layer 1 show different spontaneous astrocytic Ca^2+^ dynamics than those from layer 2/3; for instance, the average frequency of somatic Ca^2+^ events is higher in layer 1 than in layer 2/3, and the magnitude of those Ca^2+^ responses differ (Takata and Hirase, 2008); however, astrocytic membrane potential was similar for all layers (Mishima and Hirase, 2010). Hence, the diverse territorial volume of cortical astrocytes and particular Ca^2+^ dynamics at different layers might differentially influence the surrounding synapses, yielding layer differences in astrocyte–synapse interactions, ultimately establishing functional heterogeneity through the modulation of glutamate/GABA clearance and the release of active substances that affect synaptic transmission and plasticity.

Surprisingly, such layer-specific distribution is dictated by neuronal migration during development. Indeed, the layer-specific orientation of neocortical astrocytes depends on reelin (Lanjakornsiripan et al., 2018), a protein secreted predominantly from Cajal-Retzius neurons located in layer 1 that regulates the migration of cortical neurons (D’Arcangelo et al., 1995; Katsuyama and Terashima, 2009). This indicates that the existence of neuronal layers is a requirement for establishing layer-specific features of mature cortical astrocytes (Lanjakornsiripan et al., 2018). Furthermore, signaling of the neuron-derived sonic hedgehog (Shh) protein also regulates the molecular and functional profile of astrocytes across different brain regions (Farmer et al., 2016). Hence, Shh signaling in cerebellar Bergman glia promotes glutamatergic signals, enhancing expression of GLTs (GLAST) and AMPA receptors; additionally, potassium homeostasis (Kir4.1) might be related to the dense glutamatergic inputs onto Purkinje cells in the molecular layer. In contrast, cortical and hippocampal astrocytes use Shh signaling for preferential regulation of Kir4.1 channels (Farmer et al., 2016), which are related to potassium buffering. Therefore, such astrocyte regionalization seems to be dictated not only by endogenous astrocytic molecular programs, but also by neuronal signals during development. Thus, neuron–astrocyte signaling dynamically cooperates to generate astrocyte heterogeneity, and ultimately guarantees that mature astrocytes are appropriately specialized to fit the requirements of particular neural circuits.

It is important to note that astrocyte diversity might get even more complex across species. Critical molecular and anatomical differences have been found between rodent and human astrocytes (Oberheim et al., 2009; Zhang et al., 2016; Vasile et al., 2017). As an example, while a single rodent astrocyte can cover up to 120,000 synapses, a human astrocyte might cover from *ca*. 270,000 to 2 million synapses within a single domain (Bushong et al., 2002; Oberheim et al., 2009). Consequently, it is tempting to speculate that astrocytic changes in channel or transporter expression, GTs or extension of astrocytic domains will deeply impact synapses. Astrocytes by enhancing or decreasing synaptic strength would regulate the operational capabilities of human neuronal networks, and might contribute to the higher functions of the human brain.

**Figure 1:**
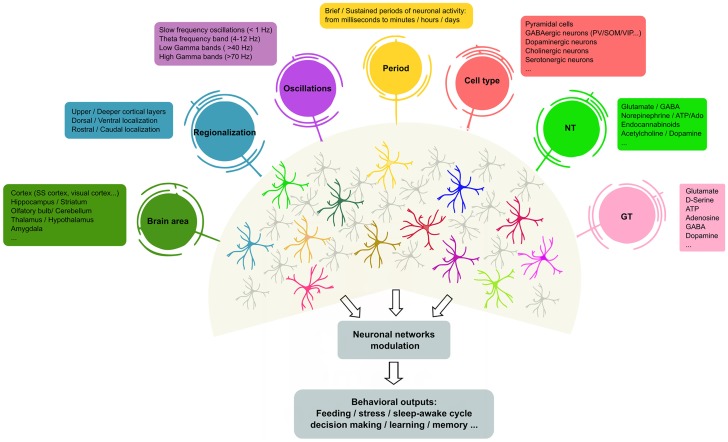
Heterogeneity of astrocyte–neuron networks. Astrocytes are able to respond to different NTs, cell types in different brain areas and under diverse patterns of stimulation. In turn, astrocytes modulate the activity of ion channels and neurotransmitter transporters in their membranes, but also release different active substances (so-called GTs, e.g., glutamate, gamma-aminobutyric acid (GABA), adenosine and D-serine, among others) that contribute to shape neuronal activity. However, it remains unclear whether the same or different astrocyte subtypes mediate these differential effects. The scheme illustrates recent evidence of the molecular and functional diversity of astrocytes that may underlie the outcomes of brain activity. In this context, single astrocytes can be affected by more than one physiological feature (represented as colored circles that connect to astrocytes), or subsets of astrocytes may show preferential links with particular physiological features, suggesting the existence of different astrocyte populations that exhibit specific properties (astrocyte color code). NTs, neurotransmitters; GTs, gliotransmitters; SS, somatosensory; PV, parvalbumin positive cells; SOM, somatostatin positive cells; VIP, vasoactive-intestinal peptide.

Collectively, these recent data indicate that astrocytes are not a homogeneous cell type, but rather are circuit-specialized cells that allow for focused astrocyte–synapse signaling, with critical consequences for information-coding in particular layers, circuits and regions in the adult brain (see Figure 1). Such astrocyte heterogeneity also provides new variables to the operational codes used by neural circuits that govern complex behavioral responses in health and disease. Therefore, our current knowledge of astrocyte physiology and its impact in synaptic function supports the idea that neuron–glia networks are complex systems that are regionally regulated, with particular structural and functional features.

## Conclusions

The aim of this review article was to provide an update on the central components that underlie the heterogeneity of astrocyte–neuron signaling, which supports the wide range of functional consequences of astrocytes on synaptic transmission and behavior. Current data show that astrocytes, via expression of ion channels, neurotransmitter receptors, subcellular Ca^2+^ dynamics, GTs release and structural changes of the cell body and fine processes, critically contribute to shape neuronal transmission. However, the full scenario of what particular features trigger molecular, structural and functional changes in astrocytes is unknown. Yet, future studies applying new approaches and methodology are required to reveal the precise mechanisms that rule astrocyte heterogeneity in different brain regions, which help to address some open questions in the field: (i) which features of astrocyte physiology are driven by neuronal activity and which others are inherent to astrocytes? (ii) what are the boundaries of brain homeostasis? That is, to what extent astrocytes can adapt themselves to neuronal changes to keep brain homeostasis; and vice versa, to what extent synapses can adapt themselves to astrocytic changes. These aims emphasize whether it is considered that altered balance of astrocyte–neuronal signaling might underlie numerous neuropathological states (AD, Huntington disease, epilepsy, major depression; for review see Lundgaard et al., 2014; Chung et al., 2015b; Koyama, 2015). Therefore, a deeper knowledge of astrocyte physiology and astrocyte–neuron networks is necessary to reveal the dynamic and complex organization of the brain circuits underlying animal behavior in health and disease.

## Author Contributions

SM, CG-A and GP wrote the article. All authors discussed the manuscript and approved the submitted version.

## Conflict of Interest Statement

The authors declare that the research was conducted in the absence of any commercial or financial relationships that could be construed as a potential conflict of interest.
